# Evaluation of a Novel Infrared Thermography Projection to Assess Udder Health in Primigravid Dairy Heifers

**DOI:** 10.3390/ani12233410

**Published:** 2022-12-03

**Authors:** Patrícia B. A. Simões, Lorenzo Viora, Pieter T. Pepler, Timothy Geraghty, Dominic J. McCafferty, Ruth N. Zadoks

**Affiliations:** 1 Department of Veterinary Medicine, University of Cambridge, Cambridge CB3 0ES, UK; 2 School of Biodiversity, One Health and Veterinary Medicine, University of Glasgow, Glasgow G61 1QH, UK; 3 Scotland’s Rural College, Veterinary Services, Craibstone Estate, Aberdeen AB21 9YA, UK; 4 Sydney School of Veterinary Science, Faculty of Science, The University of Sydney, Camden, NSW 2570, Australia

**Keywords:** infrared thermography, udder, dairy heifer, primigravid, ventrodorsal projection

## Abstract

**Simple Summary:**

Mastitis (inflammation of the mammary gland) is one of the most prevalent diseases affecting dairy cattle, and is generally diagnosed using milk samples. Pre-partum mastitis in heifers is frequently overlooked because their milk cannot be tested. Because mastitis is often characterized by elevated skin temperature, a non-invasive and contactless pre-partum screening of udder surface temperature might allow the early detection of mastitis in heifers. We demonstrate that a single ventrodorsal thermal image, which is easy and safe to collect, provides information about udder skin temperature. Therefore, it may be useful for an automated pre-partum mastitis screening system.

**Abstract:**

Heifer mastitis in early lactation impacts negatively on animal welfare, milk production and longevity. A major challenge for the prevention and control of mastitis in dairy heifers is to establish when intramammary infection occurs because pre-partum secretum sampling is risky. We evaluated a ventrodorsal projection to capture thermal images of the entire udder of primigravid and compared results against caudocranial projection, which is used in lactating cattle. Based on the analysis of 119 heifers and images taken at 2 months and 2 weeks pre-partum, a very strong positive correlation (*r* = 0.91 and *r* = 0.96, respectively) was shown between caudocranial and ventrodorsal projections of hind quarters. Quarter maximum gradient temperatures were consistently greater on ventrodorsal projection than on caudocranial projection, and less variable than minimum gradient temperatures. The collection of ventrodorsal images is a simple one-step method involving the imaging of the entire udder in a manner safe for both the cattle and handlers. Together, these results demonstrate that a single projection can be used to scan the entire udder of primigravid dairy heifers in commercial farm conditions, with the potential to implement this as a routine method for the early detection of intramammary infection based on udder surface temperature.

## 1. Introduction

Mastitis, inflammation of the mammary gland, is one of the most important diseases in dairy farms. It is a costly disease [1,2] that affects milk quality [3], animal welfare and longevity in the herd [4,5], and is generally caused by intramammary infection (IMI). Quarter-level IMI prevalence in heifers varies from 29% to 75% pre-partum, and from 12% to 57% immediately postpartum [5]. Hence, the probability of heifers starting their lactation with an unhealthy udder, with suboptimal production in terms of quantity or quality, is very high [6]. This threatens their productive performance not only during the first lactation but also in subsequent lactations [5,7], preventing heifers from fully expressing their genetic potential and affecting farms’ profitability and sustainability [5,8]. 

The early detection of mastitis in heifers is crucial for effective management and to reduce the impact of the disease in the herd [9,10]. To avoid opening the teat canal and inadvertently increasing the risk of pathogen entry and IMI, non-invasive diagnostics need to be pre-partum. Ideally, those methods would be rapid and automated, to allow routine use at the herd level. Infrared thermography (IRT) is a non-invasive technique used to assess the superficial temperature of animals, which is modified by local blood flow [11,12]. A healthy udder presents a uniform thermal image, reflecting equal distribution of blood supply at the skin surface [13]. Udder surface temperature follows a circadian pattern [14]. This daily variation, however, is smaller than the rise in temperature resulting from an inflammatory response [14], suggesting that differentiation between normal and abnormal temperature fluctuations is possible [15]. Heat is one of the cardinal signs of inflammation, and a temperature increase of the affected quarter may be observed during onset of mastitis in response to secretion of prostaglandin, histamine, serotonin and interleukins produced by proinflammatory cells which trigger vasodilatation of the mammary capillaries [12,14]. IRT has therefore been explored by several researchers as a technology with potential merit for early mastitis detection in dairy cows [10,14,16,17,18]. IRT has been applied and validated as a method for diagnosing mastitis in primiparous and multiparous cows [10,14,17,18,19]. For example, Polat et al. [10] detected a 2.35 °C greater udder skin temperature in quarters with subclinical mastitis, a strong positive correlation between udder surface temperature, somatic cell count and California milk test, and a sensitivity of 95.6% and specificity of 93.6% of IRT to detect subclinical mastitis. Other studies demonstrate a detectable rise in quarter skin surface temperatures after experimentally induced clinical mastitis, in comparison to the contralateral unchallenged control quarter [18,19,20]. However, IRT has not yet been applied to primigravid heifers. 

Validated IRT approaches include caudocranial or laterolateral projections to assess back quarters and front quarters, respectively [10,14,17,18,19]. The underdeveloped udder of nulliparae and the design of most heifer rearing facilities, e.g., cattle races and head yokes, make these approaches impractical for use in heifers. Thus, new approaches are required. The aim of the present study was to investigate the practicality of an IRT ventrodorsal projection to allow the capture of a representative image of the entire udder for calculation of surface temperature in primigravid dairy heifers under field conditions, and evaluates this approach in comparison with the established method, i.e., caudocranial projection. In doing so, we aim to demonstrate that IRT may be an attractive method for the routine on-farm screening of udder health in primigravid heifers. 

## 2. Materials and Methods

The study was conducted on a single commercial dairy farm of 700 lactating Holstein-Friesian cows, with home-bred replacement heifers and a history of heifers calving with subclinical and clinical mastitis. Heifers between nine and twenty-two months of age were housed in a contracted farm away from the main unit. Two months before the estimated calving date, these animals were moved to the main unit and maintained in a heifer group until three to two weeks pre-partum, when they were moved to a straw-bedded close-up/calving pen dedicated only to heifers. These animals were submitted to visual pre-partum udder screening to confirm udder development without specific assessment of udder health. One hundred and nineteen clinically healthy primigravid Holstein-Friesian heifers were recruited in their seventh month of pregnancy, from November 2014 to May 2015, using the herd management software DairyComp 305. 

A hand-held infrared camera (ThermaCAM^TM^ E300 IR, FLIR systems, Wilsonville, OR, USA) was used for the study. Calibration of the infrared camera was confirmed on-farm against a cylindrical matte blackbody with a thermometer probe introduced in the centre (Digital fridge/freezer alarm thermometer, Electronic Temperature Instruments Ltd., Worthing, Sussex, UK) at each session of IRT image collection. This blackbody was exposed to the shed ambient temperature for a minimum period of 30 min prior to IRT image collection. The thermometer probe calibration was checked against a mercury thermometer by submersion into water in a thermostatic water bath, which was submitted to an increase of temperature within the range of environmental temperatures expected in the shed. Shed relative humidity (RH) and ambient temperature were measured by a gauge placed near the heifers (Benetech LCD, Benetech, Shenzhen, China). This gauge sampled at a rate of 2.5 times per second and measured within ranges of −10 °C to 50 °C and RH 5% to 98%, with accuracy of ±1 °C and ±3% (30–95%), and resolution of 0.1 °C and 0.1%, for ambient temperature and RH, respectively. These parameters were registered immediately before and monitored until the end of IRT image collection. They were used to set the infrared camera measurement parameters at the beginning of each IRT session and a posteriori during the thermogram analysis. Additionally, the distance between the camera and heifers’ udder surface was measured by a digital laser measure (PLR 15 digital laser measure, Bosch, Gerlingen, Germany), fixed to the infrared camera at the same level as the lens. This digital laser measures in a range of 0.15 to 15 m, with an accuracy ±3.0 mm and measurement time of 0.5 s.

**Figure 1:**
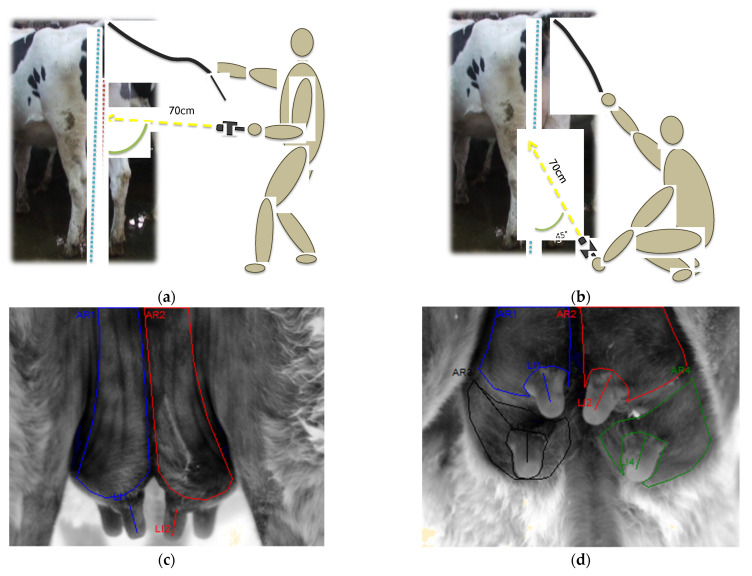
IRT projections and IRT image analysis (grey palette): (**a**,**c**) Cd-Cr—caudocranial projection; (**b**,**d**) Vt-Dr—ventrodorsal projection. AR—area defined by polygons tool; LI—line defined by lines tool. Colour area (quarter) and line (teat): blue—back left; black—front left; red—back right; green—front right.

Two sets of images were collected from each heifer at two pre-partum time points. The first set of images was collected two months pre-partum (1st IRT) and the second set two weeks pre-partum (2nd IRT). All heifers were assessed after twenty to thirty minutes of resting in a standing position, either in the cattle race or in the head yokes. To minimize the impact on animal welfare or herd management, assessments were performed prior to routine procedures, which required heifers to be restrained either in head yokes or in a cattle crush. Prior to initiating IRT image collection, digital photographs from the freeze brand and udder of each heifer were taken, in the same sequence and projections that were planned for IRT images, using a digital camera (Finepix S5500, Fujifilm, Tokyo, Japan). These were used afterwards as a visual aid for animal identification and during assessment of IRT images. The protocol included two projections of the udder for each set of IRT images: caudocranial and ventrodorsal. The caudocranial projection was taken with the camera perpendicular to the hind quarters and dropping lines from the ischial tuberosity passing via the calcanean tuber to the floor. Ventrodorsal projection was taken with an 45° angle with the dropping line ischial tuberosity-calcanean tuber-floor (Figure 1a,b). Both projections were taken at a distance of 70 cm from the udder (PLR 15 digital laser measure, Bosch, Germany). All IRT images were taken between 11 am and 2 pm, to limit the effects of the circadian rhythm. Emissivity of the thermal camera was set at 0.96, and all image collection was undertaken indoors, without wind drafts, reflective surfaces or direct sunlight interference. Rectal temperature was selected as method to assess core temperature. For each heifer, rectal temperature was taken immediately after collection of IRT images using a digital thermometer (Veterinary digital equine thermometer, Henry Schein, Kent, UK). 

The single best image from each projection from each set of images (1st and 2nd IRT), defined as the image with better contour definition in grey palette and udder position, was analysed using ThermaCAM^TM^ Researcher Pro 2.10 (FLIR System, Danderyd, Sweden) software. Environmental influences between the camera lens and udder surface were corrected within analysis software with adjustments for ambient temperature, humidity and distance to the udder. Reflective temperature was set at ambient temperature. Different geometric software-tools were used for image analysis using the grey palette. Polygons were applied to individual quarters (Figure 1c,d). Polygons were measured using manual tracing with a digital tablet for drawing (Intuos Pro creative pen tablet, Wacom, Saitama, Japan). The manual tracing of areas was conducted internally at approximately ±3 mm from the anatomical area contour to avoid the area of higher curvature. For the identification of individual quarters, intermammary grooves were taken as references. The lines were applied to individual teats (Figure 1c,d). The descriptive parameters, such as minimum (‘min’), maximum (‘max’), average (‘avg’), difference between maximum and minimum (‘max-min’) and standard deviation were obtained. All images (*n* = 470) were analysed by the same person. 

Data analysis was performed in R Version 4.2.0 [21]. Quarters were considered the unit of analysis based on the separate (patho) physiological status of individual quarters [22]. Statistical analysis focused on the descriptive parameter ‘maximum temperature’ (‘max’) and ‘minimum temperature’ (‘min’) from the area as defined by the polygons tool for quarter and from the line defined by the lines tool for teats. Temperature gradients were used in the analysis to account for any heat loss by radiation [23,24]. The temperature gradient (dT) between surface temperature (T_s_) and ambient air temperature (T_amb_) was calculated according to dT = T_s_ − T_amb_ for each udder part [23,24,25,26]. Descriptive statistics and graphical assessments were carried out for the variables in the study. Histograms and Q–Q plots were constructed, skewness and kurtosis values were calculated, and Kolmogorov–Smirnov tests with Lilliefors significance correction were performed to check normality of the data distribution, with *p* > 0.05 considered an indication of normality. Homogeneity of variances was checked using Levene’s test. Median and interquartile range were preferred to describe IRT data due to their robustness to outliers and non-normal data distributions. Means and standard errors were preferred to describe days pre-partum, ambient temperature, relative humidity and rectal temperature. The relationship between measurements obtained with the caudocranial and ventrodorsal IRT projections of 1st and 2nd IRT sets was assessed using a Pearson’s product–moment correlation. The agreement between the measurements obtained with the two projections was assessed using Bland–Altman plots [27]. Bias was defined as the mean of the dT differences between the two projections, and the standard deviation of these dT differences was calculated to assess the variability between the two projections and calculate normal approximation confidence intervals. Wilcoxon signed-rank tests, with a 95% confidence level, were used to assess the statistical significance of temperature differences between projections. Outliers were considered genuine data points, and therefore kept in the analysis. 

## 3. Results

In total, 27 IRT sessions were completed: 6 sessions two months pre-partum (1st IRT at 63.81 ± 0.53 days pre-partum, *n* = 119) and 21 sessions two weeks pre-partum (2nd IRT at 16.91 ± 0.26 days pre-partum, *n* = 116). Three heifers were missing at the time of the 2nd IRT, one animal had aborted, one was euthanized after injury, and one was sick. All sessions were synchronized with the farm routines making the procedure easy, fast and non-stressful for the heifers. 

Ambient temperature varied from 1.5 °C to 11.9 °C at 1st IRT and from 3.2 °C to 17.6 °C at 2nd IRT, with means (±*SE*) of 7.44 ± 0.31 °C and 9.03 ± 0.35 °C, respectively. Relative humidity ranged from 38.9% to 68.3% (mean 54.0 ± 0.38%) and from 24.0% to 58.2% (mean 44.2 ± 0.46%) at 1st and 2nd IRT, respectively.

Core temperatures were within the thermoneutral zone for adult cattle, 38.82 ± 0.04 °C two months pre-partum and 38.59 ± 0.03 °C two weeks pre-partum. Values outside of the thermoneutral range were reported in five animals at 1st IRT and one animal at 2nd IRT without the detection of any sign of disease. 

Quarter-level and teat-level surface temperatures and respective temperature gradients for both projections and time of IRT image collection are presented in Table 1. Wilcoxon signed-rank test showed that surface temperatures and temperature gradients on ventrodorsal projection were greater than caudocranial projection at both IRT time points. 

The Vt-Dr dT_‘max’_ values were significantly greater than those for Cd-Cr, on average by 0.6 °C (*p* < 0.001) and 0.7 °C (*p* < 0.001) for quarters at the 1st and 2nd IRTs, respectively, and by 2.4 °C (*p* < 0.001) and 1.0 °C (*p* < 0.001) for teats at the 1st and 2nd IRT, respectively. Standard deviations of these differences in quarters (1st IRT: 1.1 °C; 2nd IRT: 1.0 °C) show that, generally, the maximum temperature measured with the Vt-Dr projection were within 2 °C of the Cd-Cr measurement. The variability of dT_‘max’_ values in teats was greater than in quarters, in particular for the 1st IRT. Vt-Dr dT_‘max’_ values in teats for the 1st IRT were within 4.3 °C of the Cd-Cr measurements, and for the 2nd IRT within 2.4 °C. 

For the minimum gradient temperature, again, average Vt-Dr dT_‘min’_ values were significantly greater than those of the Cd-Cr projection, by 1.6 °C (*p* < 0.001) and 1.8 °C (*p* < 0.001) for quarters at the 1st and 2nd IRTs, respectively, and by 0.7 °C (*p* < 0.001) and 0.7 °C (*p* < 0.001) for teats at the 1st and 2nd IRTs, respectively. Further, the differences between projections in quarter measurements were more variable than those in teat measurements. The Vt-Dr dT_‘min’_ values for quarters were generally less than 6.9 °C different from Cd-Cr at the 1st IRT, and less than 5.3 °C different at the 2nd IRT. For teats, the Vt-Dr dT_‘min’_ values were less than 3.7 °C different from Cd-Cr at 1st IRT, and less than 3.3 °C at 2nd IRT.

**Figure 2:**
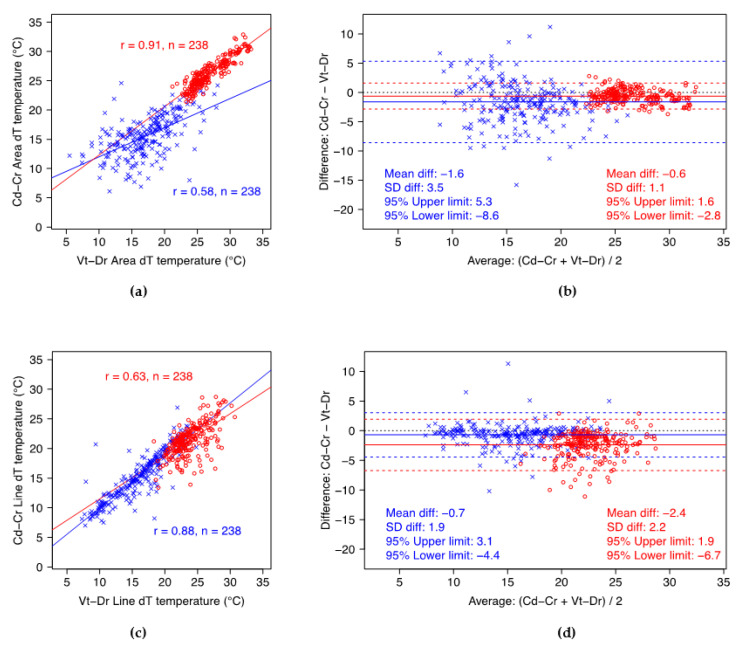
Scatterplots summarizing Pearson’s correlation (*r*) between projections (left side) and Bland–Altman plots (right side) for quarters (top row—(**a**,**b**)) and teats (bottom row—(**c**,**d**)) at 1st IRT. Cd-Cr—caudocranial projection; Vt-Dr—ventrodorsal projection; dT—temperature gradient; ∈—‘min’ temperature gradient; o—‘max’ temperature gradient.

**Figure 3:**
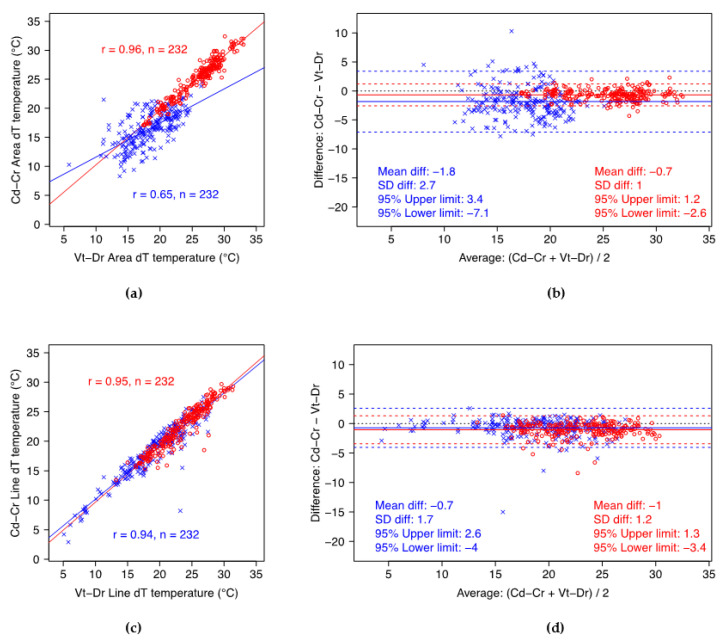
Scatterplots summarizing Pearson’s correlation (*r*) between projections (left side) and Bland–Altman plots (right side) for quarters (top row—(**a**,**b**)) and teats (bottom row—(**c**,**d**)) at 2nd IRT. Cd-Cr—caudocranial projection; Vt-Dr—ventrodorsal projection; dT—temperature gradient; ∈—‘min’ temperature gradient; o—‘max’ temperature gradient.

Overall, there were strong to very strong positive correlations between the temperature gradients of the caudocranial and the ventrodorsal projections of quarters and teats, as summarized in the scatterplots (Figure 2 and Figure 3). The two projections generally showed stronger correlations on dT_‘max’_ than on dT_’min’_, with exception of the dT_’min’_ values for teats at 1st IRT. The correlation of dT_‘min’_ between projections was stronger for teats than for quarters.

## 4. Discussion

The future production of dairy farms depends on the capacity to maintain healthy udders in both milking cows and replacement heifers. Early detection of udder health problems is important to diminish their negative impacts on herd performance [5,7,8]. A tool that allows the screening of udders of heifers, i.e., the future of the farm, before they enter the milking herd is very attractive, particularly when accomplished through a non-invasive, contactless, stress-free procedure and with automated, easy-to-read outputs. IRT has many of these desirable characteristics [28] and has the potential for automation. However, the cost of this technology in the past was one of the main hindrances to its wider use [29]. In this study, IRT images were collected during the pre-colostrogenesis and colostrogenesis phases (two months and two weeks pre-partum, respectively), representing high risk periods for IMI [30,31], which may manifest at calving or in early lactation as clinical or subclinical mastitis.

IRT has been widely used in cattle, and with known benefits in assessment of udder health in adult dairy cows [10,14,17,18,19]. Most of the IRT studies in cattle used a caudocranial projection to assess back quarters and lateral projection to assess front quarters [10,14,17,18,20,32,33]. However, those studies included primiparous and multiparous cows with larger udders than nulliparous cattle, i.e., easier to assess laterally. A lateral projection might be desirable in lactating cattle because it evaluates the lateral blood vessels, but would not be practical in heifers due to the size of the udder and the facilities available for animal handling on commercial dairy farms. In this study, a ventrodorsal projection overcame the limitations caused by the small size of the immature mammary gland at time of 1st IRT and on-farm handling system used at time of 2nd IRT, i.e., head yokes with heifers side-by-side. This approach has not been reported in dairy cattle. A similar approach was reported in dairy sheep; however, it involved sheep tipping [34]. The ventrodorsal projection shows potential to assess maximum surface temperature of quarters at both times of IRT collection (Figure 2 and Figure 3). The practical relevance of this finding is that the projection can easily be implemented on a farm, in the floors of either cattle crushes, races or milking parlours. An advantage of the ventrodorsal approach of the udder is that it allows the capture of a representative IRT image of all quarters in a single projection without disturbing the animal, which is particularly important in heifers to avoid premature or delayed calving.

Overall, Vt-Dr gradient temperatures were significantly greater than on Cd-Cr projection. This might be related to IRT reading interference due to greater proximity of the medial aspect of the hindlimbs on the lateral aspect of the udder [32]. However, this increase in temperature readings was similar for quarters in both times of IRT image collection, 1st and 2nd IRTs, for dT_’max’_ and dT_’min’_, with exception of the teats at the 1st IRT that presented a greater average difference between the dT_’max’_ of both projections. For a mastitis screening tool, quarter skin surface temperatures are of greater interest than teat temperatures. Although minimum temperature was considered, this can be biased by cooledge effects as an artefact of measurement [35]. Maximum temperature would be preferred for on-farm alerts as an increase in skin temperature is also one of the characteristics of inflammatory processes [18]. Maximum temperature was previously reported to be the best to detect changes in udder skin surface temperature caused by mastitis [32]. The selection of dT_’max’_ as the parameter for udder screening is also the choice for the Vt-Dr projection, based on the smaller variability in Vt-Dr dT_’max’_ than in Vt-Dr dT_’min’_ for quarters (Figure 2 and Figure 3). 

A polygon tool was considered the best geometric tool to detect significant udder surface temperature changes in cattle with experimentally induced *Escherichia coli* mastitis [32]. Based on this information and on the aim of covering a larger area of the udder skin, the polygon tool, rather than the line tool, was used for the analysis of quarters in IRT images. Due to the shape of the teats, the line tool was most appropriate to avoid curvature effects on surface temperature. Ideally, the analysis process would be simplified by automated evaluation with analytical software with algorithms that recognize udder contours and exclude border areas and intermammary grooves [32], as used for the detection of breast cancer in women [36]. Careful consideration of these aspects may help us to reduce errors in udder surface temperature measurements due to udder curvature, proximity to the medial aspect of the hindlimbs and shape of the intermammary groove, and to reduce effort in analysing images [32,35]. The manual analysis of IRT images is a feature that limits its usefulness for a routine on-farm application. To be attractive for use by farmers, automation is likely to be the next step for mastitis screening. 

Despite the increased use of IRT technology in veterinary medicine, little information is available on its use in field conditions to detect mastitis and no reports were found in the udder screening of primigravid dairy heifers. IRT studies in field conditions are difficult due to environmental factors that can affect temperature readings, such as ambient temperature, humidity, wind and exposure to sunlight [37,38]. The impact of these factors was considered and minimised by our study protocol, which could be accommodated in commercial dairy farms or rearing units. Images were collected in a routine handling area with no reflective surfaces. The indoor location used for image collection was protected from wind drafts; therefore, air velocity was considered low and, consequently, low heat exchange by convection could be assumed [25]. Direct exposure to sunlight (especially important in heifers with dark pigmented udder skin), intense exercise and brushing of the udder skin were avoided due to their potential to increase udder surface temperature, whereas wind drafts and wet udder skin could decrease udder surface temperature. A constant distance (<1 m) between the udder surface and lens of the thermal camera was maintained to minimise the atmospheric absorption of IR [39]. The angle of the camera lens with the udder surface at 45 degrees has previously been shown not to influence thermal readings [39]. IRT image collection was performed at the same time of the day on each occasion to minimise the effect of circadian rhythm on udder surface temperature, which can lead to a variation of 0.34 °C [40,41]. Additionally, all image collection was performed after 20 to 30 min of heifers being handled and prior to any other data collection. To fulfil these requirements, automated ventrodorsal IRT measurements could be conducted at the entrance of a milking parlour or in an in-house cattle race, after routine animal handling and a resting period at the collecting yard. This approach avoids disruption of the farm routine and does not impact on heifers’ welfare through the synchronization of udder screening with scheduled routine procedures. In most UK dairy farms, hair removal from the udder in lactating cattle is a routine procedure; however, this is rarely performed in primigravid heifers. Therefore, in this study, the hair coat of the udder was not removed. The hair coat was not brushed or washed, in part to avoid effects on measurements, but also because of the very low degree of dirt on the study farm, and to avoid practical difficulties associated with hair removal from primigravid udders. Hair density and coat type does influence surface temperature measurement [42] and should be considered in future studies. 

Our results can be considered as a validation of IRT ventrodorsal projection as a method to assess udder surface temperature. However, due to the absence of clinical mastitis cases, it was not possible to assess IRT as a diagnostic tool; therefore, further studies are required to understand its usefulness as diagnostic screening tool in on-farm settings.

## 5. Conclusions

IRT allows the collection of udder surface temperatures in a fast, non-invasive, non-stressful and contactless manner. Ventrodorsal projection of the udder is an alternative to craniocaudal projection and might be preferable in the screening udders of primigravid heifers before calving. Installed on the floors of cattle crushes, races or milking parlours, automated IRT technology could allow the detection of variations in temperature of individual quarters or of the overall udder surface, even in the underdeveloped udders of heifers. However, further on-farm studies and advances in image analysis software are needed to validate its usefulness for mastitis diagnosis and to enable its use in commercial settings.

## Figures and Tables

**Table 1:** Descriptive results of surface temperature (Ts) and temperature gradient (dT) at first (*n* = 119 heifers) and second IRT (*n* = 116 heifers).

			Temperature (°C)		
IRT	Udder Region	Projection		Median [Q1–Q3]	IQR
1st IRT	Quarter	Cd-Cr	Ts_‘min’_	23.55 [20.4–26.0]	5.6
			Ts_‘max’_	34.00 [32.5–34.7]	2.2
			dT_‘min’_	15.70 [13.3–17.9]	4.6
			dT_‘max’_	25.95 [24.5–28.0]	11.5
		Vt-Dr	Ts_‘min’_	25.25 [22.2–27.8] *	5.6
			Ts_‘max’_	34.50 [33.5–35.2] *	1.7
			dT_‘min’_	17.00 [14.5–19.9] *	5.4
			dT_‘max’_	26.20 [24.7–29.1] *	4.4
	Teat	Cd-Cr	Ts_‘min’_	23.20 [19.9–26.4]	7.2
			Ts_‘max’_	29.30 [27.1–31.5]	4.4
			dT_‘min’_	15.10 [12.2–18.0]	5.9
			dT_‘max’_	21.40 [19.8–23.1]	3.3
		Vt-Dr	Ts_‘min’_	24.05 [19.9–27.5] *	7.7
			Ts_‘max’_	31.05 [29.8–33.0] *	3.2
			dT_‘min’_	16.40 [12.8–18.7] *	5.9
			dT_‘max’_	23.40 [22.2–25.5] *	3.3
2nd IRT	Quarter	Cd-Cr	Ts_‘min’_	25.95 [23.6–28.2]	4.6
			Ts_‘max’_	34.40 [33.5–34.9]	1.4
			dT_‘min’_	16.70 [14.4–18.8]	4.4
			dT_‘max’_	25.75 [22.0–27.5]	5.5
		Vt-Dr	Ts_‘min’_	28.10 [25.8–29.7] *	3.9
			Ts_‘max’_	36.80 [34.4–35.6] *	1.2
			dT_‘min’_	18.50 [15.7–21.2] *	5.5
			dT_‘max’_	26.70 [23.0–28.5] *	5.5
	Teat	Cd-Cr	Ts_‘min’_	28.80 [26.9–30.1]	3.3
			Ts_‘max’_	32.10 [30.8–33.0]	2.2
			dT_‘min’_	19.25 [15.6–21.6]	6.1
			dT_‘max’_	22.80 [19.2–25.5]	6.3
		Vt-Dr	Ts_‘min’_	29.30 [27.8–31.9] *	3.0
			Ts_‘max’_	33.10 [30.7–33.8] *	1.9
			dT_‘min’_	19.70 [16.3–22.5] *	6.2
			dT_‘max’_	23.75 [20.7–26.3] *	5.6

Cd-Cr—caudocranial; Vt-Dr—ventrodorsal; IQR—interquartile range; Q1—first quartile and Q3—third quartile: Tukey’s hinges; ‘min’—minimum; ‘max’—maximum; * significant difference between temperature at Vt-Dr and correspondent Cd-Cr projection (*p* < 0.001).

## Data Availability

The data set generated for this study can be found in the Apollo DOI 10.17863/CAM.91291.
